# Effects of Prostaglandin E1 and Balloon Atrial Septostomy on Cerebral Blood Flow and Oxygenation in Newborns Diagnosed with Transposition of the Great Arteries

**DOI:** 10.3390/biomedicines12092018

**Published:** 2024-09-04

**Authors:** Manuela Cucerea, Maria-Livia Ognean, Alin-Constantin Pinzariu, Marta Simon, Laura Mihaela Suciu, Dana-Valentina Ghiga, Elena Moldovan, Mihaela Moscalu

**Affiliations:** 1 Neonatology Department, GEP University of Medicine Pharmacy, Science and Technology of Targu Mures, 540142 Târgu Mureș, Romania; manuela.cucerea@umfst.ro (M.C.); marta.simon@umfst.ro (M.S.); lauramihaelasuciu@yahoo.com (L.M.S.); 2 Dental Medicine and Nursing Department, Faculty of Medicine, Lucian Blaga University of Sibiu, 550169 Sibiu, Romania; 3 Department of Morpho-Functional Sciences II, “Grigore T. Popa” University of Medicine and Pharmacy, 700115 Iași, Romania; alin.pinzariu@umfiasi.ro; 4 Faculty of Dental Medicine, GEP University of Medicine Pharmacy, Science and Technology of Targu Mures, 540139 Târgu Mureș, Romania; valentinaghiga@gmail.com; 5 Pediatric Intensive Care Unit, Cardiovascular and Transplant Emergency Institute, 540136 Târgu Mureș, Romania; bitirelena@gmail.com; 6 Department of Preventive Medicine and Interdisciplinarity, “Grigore T. Popa” University of Medicine and Pharmacy, 700115 Iași, Romania; moscalu.mihaela@gmail.com

**Keywords:** dextro-transposition of the great arteries, prostaglandin E1, septostomy, crSO_2_, cFTOE, cerebral doppler ultrasound

## Abstract

Dextro-transposition of the great arteries (D-TGA) is a critical congenital heart defect that can impact neurodevelopment due to cerebral perfusion and oxygenation disorders followed by alterations in synaptogenesis, gyrification, sulcation, and the microstructure. Brain injuries can occur both pre-operatively and postoperatively, especially white matter injuries, neuronal loss, and stroke. *Materials and Methods*: In a retrospective study conducted at a tertiary center between 2016 and 2023, we investigated the early effects of Prostaglandin E1 (PGE1) administration and balloon atrial septostomy (BAS) on cerebral blood flow and oxygenation in inborn neonates with D-TGA. Cerebral Doppler Ultrasound in the anterior cerebral artery (ACA) was performed to assess the resistive index (RI), Peak Systolic Velocity (PSV), and End-Diastolic Velocity (EVD) before PGE1, before the BAS procedure, and 24 h after birth. Cerebral regional saturations of oxygen (crSO_2_) and cerebral fractional tissue oxygen extraction (cFTOE) were evaluated. D-TGA patients were divided into the PGE1 group and the PGE1 + BAS group. Age-matched healthy controls were used for comparison. *Results*: All 83 D-TGA newborns received PGE1 within two hours after delivery, of whom 46 (55.42%) underwent BAS. In addition, 77 newborns composed the control group. PGE1 administration increased crSO_2_ from 47% to 50% in the PGE1 group, but lower than in controls at 24 h of life, while cFTOE remained elevated. The RI increased 24 h after delivery (0.718 vs. 0.769; *p* = 0.000002) due to decreased EDV (10.71 vs. 8.74; *p* < 0.0001) following PGE1 treatment. The BAS procedure resulted in a significant increase in crSO_2_ from 42% to 51% at 24 h of life in the PGE1 + BAS group. Doppler parameters exhibited a similar trend as observed in the PGE1 group. *Conclusions*: PGE1 treatment and BAS are lifesaving interventions that may improve cerebral perfusion and oxygenation in newborns with D-TGA during the transition period, as reflected by increasing SpO_2_ and crSO_2_.

## 1. Introduction

Dextro-transposition of the great arteries (D-TGA) is a critical congenital heart defect that affects a significant number of newborns, with a prevalence of 1 to 47 cases per 10,000 births. In this condition, the pulmonary artery and the aorta are switched, but the atrioventricular concordance is maintained [1,2]. This results in a lethal hemodynamic pattern where both systemic and pulmonary circulations run in parallel, causing severe hypoxemia in the brain and vital organs, deep acidemia, and a low cardiac output. Survival crucially depends on mixing oxygenated and deoxygenated blood through the atrial septal defect (ASD or persistent foramen ovale—FO), ventricular septal defect (VSD), or patent ductus arteriosus (PDA) [3]. The spontaneous closure or restriction of these defects at birth significantly increases the risk of severe cyanosis and acute cardiorespiratory deterioration [4]. Dextro-transposition of the great arteries can be classified as simple if the ventricular septum is intact or complex if it is accompanied by other congenital heart defects, such as a ventricular septal defect (VSD), left ventricular outflow tract obstruction, or coarctation of the aorta [5,6].

The clinical presentation of D-TGA depends on the mixing of blood through additional communications [7], resulting in cyanosis that is unresponsive to oxygen supplementation, isolated tachypnea, and occasionally a cardiac murmur associated with PDA or ASD/VSD [8]. This condition can be accurately diagnosed through either a prenatal or postnatal echocardiogram [9].

The arterial switch is the gold-standard surgical approach for treating D-TGA. After birth, immediate management includes ensuring blood mixes between the systemic and pulmonary circulations. Prostaglandin E1 (PGE1) is administered intravenously to maintain a patent ductus arteriosus. If PGE1 treatment is ineffective and the newborn is unstable, or if the ASD/PFO is restricted, it is crucial to perform a Raskind atrial balloon septostomy (BAS), ideally within the first 24 h of life [10,11].

The cardiac anomalies in D-TGA may alter the cerebral blood flow (CBF) and oxygenation even before birth, leading to ischemic and hypoxic cerebral injuries and long-term neurodevelopmental impairments [12]. During prenatal development, an abnormal heart anatomy and physiology can disrupt the supply of nutrients and glucose to the brain. This, along with hypoxia and CBS modification, leads to alterations in synaptogenesis, gyrification, sulcation, and the microstructure, ultimately resulting in a smaller brain size at birth [13,14]. Brain injuries can occur both preoperatively and postoperatively, especially white matter injuries and stroke [14,15]. Preoperative medications required to maintain PDA and BAS for atrial blood mixing can also contribute to these complications [16]. After birth, preoperative/intraoperative and post-surgery brain monitoring can detect alterations in the CBF and oxygenation, enabling interventions to enhance neurological outcomes [17].

Arterial spin-labeled perfusion magnetic resonance imaging (ASL-pMRI) and optical diffuse correlation spectroscopy allow for the accurate determination of CBF after birth. However, its accessibility in neonatal units or during the preoperative period is often limited by patient hemodynamic instability [18,19,20,21]. Instead, cerebral Doppler ultrasound (CDU) is a noninvasive bedside tool used to evaluate CBF intermittently and provides a real-time overview of cerebral perfusion. It assesses the blood flow velocity in the cerebral arteries during both systole and diastole and helps estimate resistance to the cerebral blood flow [22,23]. Assessing the blood flow velocity in the diastole can indicate the degree of the ductal steal phenomenon. If a retrograde diastolic flow is present, it means an early need for surgical correction [9].

Near-infrared spectroscopy (NIRS) is a method used to measure the trend of cerebral regional tissue oxygen saturation (crSO_2_) and cerebral fractional tissue extraction (cFTOE). This helps in assessing preoperative cerebral hypoxia by indirectly measuring the balance of cerebral oxygen consumption and supply [18]. NIRS values provide information about the perfusion and oxygenation status.

This study aimed to investigate the effects of emergency procedures performed before surgery on the cerebral blood flow and cerebral oxygen levels in newborns with D-TGA within the first 24 h of life. We used a cerebral Doppler ultrasound to examine the impact of initial treatments on cerebral flow velocities and NIRS technology to evaluate cerebral oxygenation. The data from newborns treated with PGE1 alone, PGE1 + BAS, and age-matched controls were compared.

## 2. Materials and Methods

This retrospective case–control study was conducted at a tertiary perinatal center in Târgu Mureș, Romania, which houses pediatric cardiology and neonatal cardiac surgery departments within the same hospital from 1 January 2016 to 31 December 2023.

We aimed to identify the changes in CBF and cerebral oxygenation after the initiation of preoperative PGE1 treatment to maintain patency of ductus arteriosus and BAS, respectively. This study was approved by the hospital’s ethics board committee (Nr.35518/27.11.2019). Written consent of the mother was obtained for this study.

### 2.1. Study Groups

Inclusion criteria: inborn neonates diagnosed pre/postnatally with D-TGA with intact ventricular septum (IVS-TGA) and D-TGA with ventricular septal defect (VSD-TGA).

Exclusion criteria: inborn neonates with complex D-TGA or with late diagnosis (after 24 h of life) and D-TGA newborns transferred to our center after 24 h of life.

**Figure 1:**
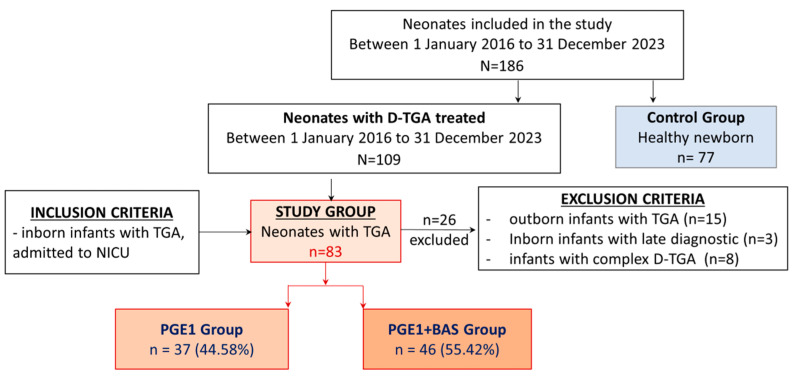
Flow chart for study groups.

Newborns with D-TGA undergoing heart surgery for the arterial switch were divided into two groups: the PGE1 group and the PGE1 + BAS group. The control group included healthy full-term newborns who were recruited from the low-risk obstetrics department (Figure 1).

### 2.2. D-TGA Management and Study Intervention

As per the unit protocol, the management of D-TGA involves initiating continuous intravenous PGE1 treatment at a starting dose of 0.03–0.05 micrograms per kilogram per minute. The dose can be gradually increased or decreased by 0.01 micrograms per kilogram per minute to achieve a peripheral oxygen saturation (SpO_2_) of 80–85% within thirty minutes to two hours. Doses should be individualized based on tolerance, and the maximum dose used is 0.1 micrograms per kilogram per minute. In cases where SpO_2_ does not improve, and other left-to-right shunts that facilitate blood mixing for oxygenation are insufficient, the pediatric cardiologist may indicate performing BAS as soon as possible after starting PGE1 infusion. Arterial switch surgery is routinely performed at our institution for patients with D-TGA aged 7 to 16 days. PGE1 doses are meticulously adjusted based on hemodynamic stability, SpO_2_ levels, and serial echocardiographic assessments.

### 2.3. Data Collection

The demographic data collected from clinical records for study groups include gender, gestational age (GA), birth weight (BW), head circumference (HC), antenatal care, antenatal diagnosis, intrauterine transfer, mode of delivery, Apgar scores (at 1 and 5 min), days between birth and arterial switch operation, and pre-and post-surgery deaths.

The clinical and ultrasound data collected include blood pressure (BP) measurement, pre-ductal oxygen saturation (SpO_2_), cerebral regional oxygen saturation (crSO_2_), cerebral fractional tissue extraction (cFTOE), FiO_2_, arterial blood gases (pO_2_, HCO_3_, lactate, and hemoglobin level), and cardiac and head ultrasounds. For D-TGA groups, we noted the presence of tachypnea, cyanosis, cardiac murmur, need for mechanical ventilation and inotropes, and PGE 1 doses.

The outcome variables included the change in pre-ductal SpO_2_, crSO_2_, cFTOE, blood gases, FiO_2_, and BP at 24 h of life after each intervention (PGE1 treatment/BAS). We noted the changes in the ACA velocities and RI at 24 h after delivery in all groups, and we assessed the size of the ASD after the BAS procedure in the D-TGA group who underwent septostomy.

### 2.4. Timing of Patient Evaluation

For the clinical and ultrasound evaluation of the patients and comparison purposes, we defined three specific time points: 1. for all study groups, the first assessment took place within the first 2 h after birth; 2. for the group that required septostomy, the second assessment occurred just before BAS procedure; and 3. the third assessment was conducted 24 h after birth for all study groups (Table 1).

### 2.5. Cardiac Ultrasounds

An echocardiogram, performed by a pediatric cardiologist, was used to evaluate the overall cardiac function, anatomy, and type of D-TGA (TGA-IVS or TGA-VSD). In the study, every newborn with D-TGA received an echocardiogram within two hours after delivery before starting PGE1 treatment. Subsequent echocardiograms were performed at 24 h of life in the PGE1 group. Additionally, an echocardiogram was conducted before septostomy and at 24 h of life in the PGE1 + BAS group, measuring ASD size (mm), ASD gradient, and PDA size (mm) post-BAS procedure. As per the unit protocol, we defined an ASD/FOP as restrictive if the size is less than 4 mm and the gradient is more than 5 mmHg.

### 2.6. Cerebral Ultrasounds

**Figure 2:**
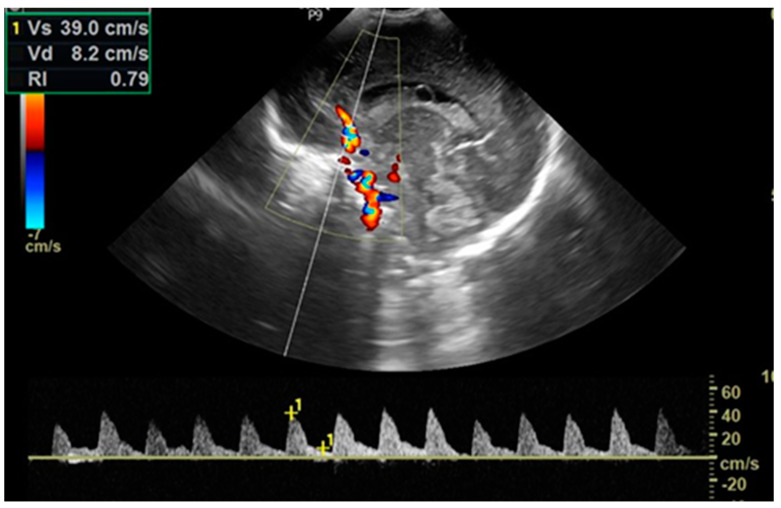
Doppler investigation of Anterior Cerebral Artery (ACA).

The cerebral ultrasound was performed using the LOGIQ e9 ultrasound machine in coronal and sagittal planes by trained neonatologists with a 7.5–12 MHz transducer. After scanning for structural abnormalities, a Doppler investigation was conducted by placing a pulsed Doppler sample volume gate at the level of the anterior cerebral artery (ACA) and positioned anterior to the genu of the corpus callosum in the sagittal view, with the insonation angle close to 0. The ACA flow velocities were assessed by measuring peak systolic velocity (PSV in cm/s) and end-diastolic velocity (EDV in cm/s) and then automatically calculating the resistive index (RI/Pourcelot index) (Figure 2). Cerebral ultrasounds were performed for every newborn in the D-TGA and control groups within two hours after delivery and again at 24 h of life. Newborns with BAS also underwent another cerebral ultrasound examination before septostomy.

### 2.7. SpO_2_ Measurement

In our practice, we continuously monitor SpO_2_ in both the right upper limb (pre-ductal) and lower limb (post-ductal) using pulse oximetry. The SpO_2_ values were measured using Nellcor (Medtronic, Dublin, Ireland) sensors. For this study, we used pre−ductal SpO_2_ for comparison, which reflects the level of oxygenation reaching the brain.

### 2.8. crSO_2_ and cFTOE Measurement

We utilized an INVOS 5100C near-infrared spectrometer (NIRS) for continuous monitoring of cerebral oxygenation (crSO_2_). Neonatal NIRS sensors were positioned on the infants’ foreheads, and cerebral fractional tissue oxygen extraction (cFTOE) was determined using the formula (SpO_2_ − crSO_2_)/SpO_2_.

### 2.9. Blood Pressure Measurement

Noninvasive blood pressure (BP) measurements were taken using the oscillometric method with a properly sized cuff on the right upper arm. BP values were recorded at specific time points. We measured blood pressure (systolic, diastolic, and mean arterial pressure (MAP)).

### 2.10. Blood Gases Measurement

The capillary blood sample was taken from the heel and analyzed with the same blood gas analyzer. pH, partial pressure of oxygen (pO_2_), and lactate were recorded and analyzed.

### 2.11. Statistical Analyses

The statistical processing of the analyzed variables was performed in SPSS v.29 (IBM Ireland Product Distribution Limited, Dublin, Ireland) and the STATA 16 software (StataCorp LLC, College Station, TX, USA). The data analysis involved presenting the parameters descriptively and applying comparison tests based on the type of variables. For continuous variables with a normal distribution, we reported the mean ± standard deviation (SD), and for variables not normally distributed, we used the median (interquartile range—IQR). The normal distribution was verified using the Kolmogorov–Smirnov test, and the homogeneity of the variables was checked with the Levene test (Homogeneity of Variance Test). To compare the parameters, we used the Kruskal–Wallis H Test for comparisons between three or more sets of values, followed by post hoc analysis or Student’s *t*-test. Additionally, depending on the variable characteristics, the Mann–Whitney U Test was applied as appropriate. The significance level (*p*-value) was considered significant for values less than *p* = 0.05.

## 3. Results

We have identified 109 patients who were diagnosed with D-TGA and received treatment in our NICU. We excluded 26 patients: eight complex D-TGA cases, three inborn D-TGA cases with late diagnosis, and 15 outborn D-TGA cases. Therefore, 83 out of 109 participants were enrolled in the study and received initial PGE1 treatment, of which 46 underwent BAS. The control group consisted of 77 healthy newborns matched for gestational age (Figure 1).

### 3.1. Baseline Characteristics

In our study, we found no significant differences in GA, BW, or antenatal care between the groups. Additionally, there were no substantial variances in the prenatal diagnosis, in utero transfer, mode of delivery, Apgar scores, age at surgery, and pre- and post-surgery mortality between the two groups with D-TGA. Conversely, the head circumference was significantly smaller in the PGE1 + BAS group compared to the control group (33.8 ± 1.3 cm vs. 34.5 ± 1 cm, *p* = 0.0151) (Table 2).

The Apgar scores at 1 and 5 min were similar between the D-TGA groups, but significantly lower than those in the control group. Furthermore, we observed a higher rate of neonatal deaths in the PGE1 + BAS group compared to the PGE1 group (17.4% vs. 2.7%, *p* = 0.0240).

### 3.2. Clinical Characteristics, Interventions, and Heart Ultrasound Evaluation

We analyzed both study groups with D-TGA based on the clinical characteristics, interventions (PGE1 treatment and BAS procedure), and heart ultrasound evaluation. The clinical variables such as tachypnea, cyanosis, cardiac murmur, and the need for mechanical ventilation and inotropes did not show significant differences between the D-TGA groups. Although the initial doses of PGE1 (μg/kg/min) were almost similar, at 24 h of life, the doses were significantly lower in the PGE1 + BAS group compared with the PGE1 group (0.031 ± 0.015 vs. 0.040 ± 0.016; *p* = 0.0136). In the PGE1 + BAS group, the mean time to BAS was 8.71 ± 5.05 h after delivery. After septostomy, the size of ASD/FOP increased from a median of 3 mm to 4.65 mm in the PGE1 + BAS group. No deaths occurred because of the BAS procedure, and no early complications related to the procedure were recorded. The results are presented in Table 3.

Regarding the characteristics of the PDA, such as the size and direction of the blood flow, we did not find any significant differences between the D-TGA groups. However, we noticed variations in left-to-right shunts that facilitate blood mixing for oxygenation. Thus, at birth, the group requiring septostomy had a significantly smaller median size of ASD/PFO (median 3 mm vs. 4.8 mm; *p* < 0.001) and a significantly higher ASD gradient (8.7 mmHg vs. 5 mmHg; *p* < 0.001). In the PGE1 + BAS group, the VSD size (0.63 ± 1.99 mm vs. 1.57 ± 2.59 mm; *p* = 0.0393) was observed to be smaller compared to the PGE1 group.

### 3.3. Effect of PGE1 and BAS on Peripheric and Cerebral Oxygenation

Within 2 h of life, before the initiation of PGE1 infusion, the median of pre-ductal SpO_2_ was significantly lower in the group treated with PGE1 and BAS compared to the group treated with PGE1 alone (median value 72% vs. 80%; *p* = 0.00012), while both D-TGA groups had lower pre-ductal SpO_2_ than the control group (median value 99%). Conversely, at 24 h of life, the median of pre-ductal SpO_2_ was significantly higher in the PGE1 + BAS group than in the PGE1 group (median value 84.5% vs. 82%; *p* = 0.00003), but it was still lower than in the control group (99%).

Patients requiring septostomy had significantly lower median crSO_2_ before initiating medical treatment, as compared to those receiving only PGE1 treatment (median value 43% vs. 47%; *p* = 0.0412). Both D-TGA study groups had significantly lower median crSO_2_ values than the control group (median value 83%; *p* < 0.001). At 24 h after birth, crSO_2_ increased in both D-TGA groups. The values were similar, but still significantly lower than those in the control group (median value 50% and 51% vs. 87%; *p* < 0.001).

The median cFTOE values in the PGE1 (median value 0.38 vs. 0.16; *p* < 0.001) and PGE1 + BAS (median value 0.39 vs. 0.16; *p* < 0.001) groups were higher than those in the control group, both before the initiation of PGE1 treatment and at 24 h of life (median value 0.38 vs. 0.11; *p* < 0.001) (Table 4).

Before starting PGE1 treatment, both D-TGA groups had a significantly higher oxygen demand compared to the control group (30% and 40% vs. 21%, *p* < 0.0001), while median pO_2_ levels were lower in the PGE1 + BAS group than the PGE1 group (median 26 mmHg vs. 31 mmHg; *p* = 0.0431). At 24 h after birth, after PGE1 treatment initiation/after BAS, the requirement for oxygen decreased significantly in both groups with D-TGA. Still, it remained higher than in the control group (*p* < 0.0001). The median pO_2_ values were similar in the D-TGA groups, but significantly lower compared to the control group (median 56 mmHg; *p* < 0.001).

Regarding blood pressure, we observed a lower mean arterial pressure (MAP) in the PGE1 group compared to the PGE1 + BAS group (43.73 mmHg vs. 49.3 mmHg; *p* = 0.0012) before PGE1 initiation. However, this difference no longer existed at 24 h of life. The mean systolic blood pressure was significantly lower in the PGE1 group compared to the PGE1 + BAS group (65.41 mmHg vs. 69.78 mmHg; *p* = 0.0147) and in the control group (65.41 mmHg vs. 73.10 mmHg; *p* = 0.00003) 24 h after birth. The mean diastolic blood pressure was also lower in the PGE1 group compared to both the PGE1 + BAS group (29.70 mmHg vs. 33.67 mmHg; *p* = 0.0140) and the control group (29.70 mmHg vs. 36.96 mmHg; *p* = 0.00004) within two hours after delivery. At 24 h of life, the mean diastolic blood pressure was similar in the two D-TGA groups, but significantly lower compared to the control group.

### 3.4. Effect of PGE1 and BAS

Before starting PGE1 infusion, the mean RI was similar in both D-TGA groups, with no significant difference compared to the control group. Instead, at 24 h after birth, newborns with D-TGA exhibited significantly higher mean RI compared to the control group (0.769 and 0.764 vs. 0.681; *p* = 0.00002), due to the significant decrease of EDV in the D-TGA groups (Table 5).

### 3.5. Comparison of Cerebral/Peripheric Oxygenation and Cerebral Velocities between Groups

**Figure 3:**
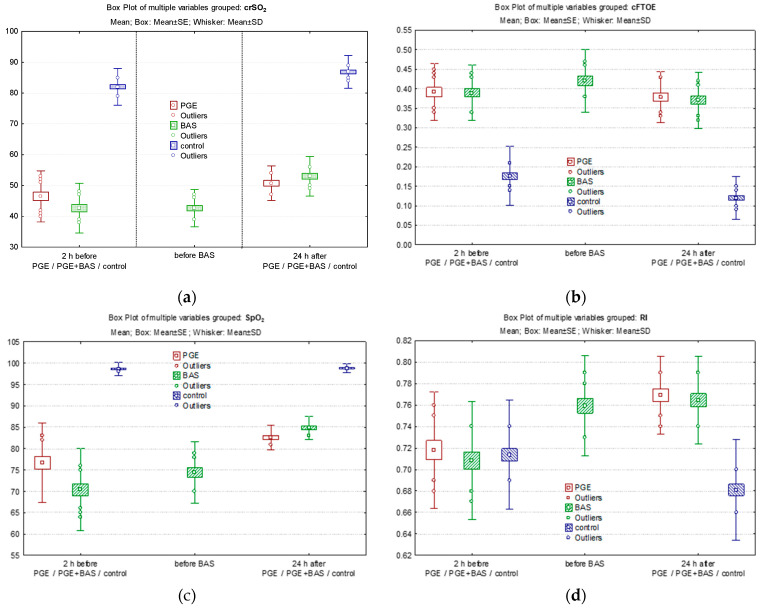

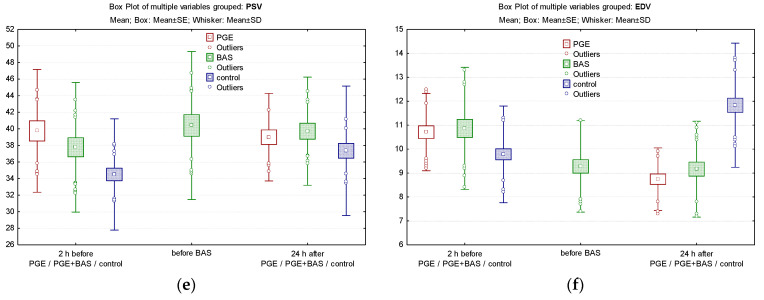
Comparison of cerebral/peripheric oxygenation and cerebral velocities between groups: (**a**) crSO_2_; (**b**) cFTOE; (**c**) SpO_2_ preductal; (**d**) RI; (**e**) PSV; and (**f**) EDV.

In the PGE1 group, following PGE1 treatment, crSO_2_ improved from a median of 47% before PGE1 to 50% at 24 h of life (Figure 3a), while cFTOE remained constant and did not change significantly (Figure 3b). SpO_2_ significantly improved from a median of 80% before PGE1 to 82% at 24 h of life (Figure 3c). The RI significantly increased 24 h after delivery (0.769 ± 0.036 vs. 0.718 ± 0.054; *p* = 0.000002) (Figure 3d) due to decreased EDV (8.74 ± 1.31 vs. 10.71 ± 1.61; *p* < 0.0001) following PGE1 treatment (Figure 3e), while SV remained unchanged (Figure 3f).

In the PGE1 + BAS group, crSO_2_ decreased from a median of 43% before the initiation of PGE1 treatment to 42% before the BAS procedure and then increased to 51% at 24 h of life following septostomy (Figure 3a). cFTOE increased before septostomy and decreased significantly at 24 h (Figure 3b). Pre-ductal SpO_2_ significantly improved, rising from a median of 72% before PGE1 to 77% before BAS and reaching 84.5% at 24 h of life (*p* < 0.0001) (Figure 3c). Doppler parameters in ACA followed the same trend as in the PGE1 group. This involved an increase in the RI (0.708 ± 0.054 vs. 0.759 ± 0.046 vs. 0.764 ± 0.041; *p* = 0.00002), a decrease in the end-diastolic velocity (10.86 ± 2.54 vs. 9.27 ± 1.91 vs. 9.16 ± 2.00; *p* = 0.00004), and maintenance of PSV within the same limits at the three specific time points of evaluation (Figure 3d–f).

## 4. Discussion

This study aimed to assess the impact of PGE1 treatment and BAS on cerebral and peripheric oxygenation and blood flow velocities 24 h after birth, as both interventions affect cerebral hemodynamics. In our study, 70.3% of patients with D-TGA who received PGE1 treatment and 63.04% of patients who were treated with both PGE1 and BAS were prenatally diagnosed and confirmed postnatally. This detection rate is consistent with findings from other studies, which typically range between 50% and 80% [24,25]. All patients were born at our center, with over 50% transferred in utero, enabling early management and preventing severe hypoxemia and hemodynamic compromise during postnatal transfer, especially in patients with inadequate intracardiac blood mixing. A study conducted by Thomas et al. revealed that infants born at a tertiary care center had a shorter median time to BAS (4 h vs. 14.1 h, *p* < 0.0001) compared to those requiring transport. A shorter time to BAS was correlated with reduced NICU and hospital stays (r = 0.21, *p* = 0.046 and r = 0.24, *p* = 0.02, respectively), but it had no impact on mortality [24]. Veal and collab. found no significant differences in outcomes between infants delivered in tertiary centers and those transferred in by a specialized team [26].

In this study, the mean age at the arterial switch was 14 ± 10.1 days in the PGE1 group and 14.1 ± 6.6 days in the PGE1+ BAS group, respectively, higher than reported in an extensive study of 1772 D-TGA patients conducted by Dorobantu et al. (9.5 days (IQR: 6.5–14.5)) [27]. The timing of surgery in the first few hours of life [28] is still a subject of debate, while current guidelines recommend surgery in the first week of life [29].

Newborns with D-TGA usually experience the delayed closure of the ductus arteriosus for a few days due to inadequate constriction stimulation caused by the low partial pressure of oxygen [30,31]. Therefore, PGE1 infusion is the standard of care in newborns with D-TGA. The goal of PGE1 treatment is to enhance hemodynamic status and oxygenation, protecting the brain from the adverse effects of hypoxemia until the arterial switch intervention [9,32]. PGE1 functions by interacting with specific G protein-coupled receptors (GPCRs) on the surface of target cells, notably the EP receptors (EP1, EP2, EP3, and EP4) [33,34]. The binding of PGE1 to these receptors stimulates intracellular signaling cascades, including the adenylate cyclase pathway, which increases cyclic AMP (cAMP) levels [34,35]. Elevated cAMP functions as a secondary messenger, activating protein kinase A (PKA). PKA, in turn, phosphorylates many target proteins, causing smooth muscle relaxation and vasodilation [36]. The vasodilator action is crucial for keeping the ductus arteriosus open to allow the proper mixing of oxygenated and deoxygenated blood. Furthermore, PGE1 has been proven to inhibit platelet aggregation and have anti-inflammatory characteristics, which adds to its therapeutic effectiveness in neonates with D-TGA [37]. PGE1 reduces the chances of severe hypoxemia and cardiac instability in diagnosed neonates by ensuring a proper blood flow and oxygen delivery. In our study, all 83 D-TGA patients were treated with PGE1 within the first two hours after birth at the mean initial dose between 0.042 and 0.05 μg/kg/min. The PGE1 infusion was adjusted based on echocardiographic assessments, peripheral oxygen saturations, and clinical signs to administer the lowest effective dose, considering its dose-dependent side effects [9]. High doses may lead to increased pulmonary circulation and worsened breathing, requiring oxygen therapy and respiratory support [38].

However, adequate interatrial communication is essential for mixing oxygenated and deoxygenated blood, and septostomy is necessary when communication is restrictive [39]. Thus, ensuring the stabilization of neonates with D-TGA before surgery significantly enhances the postoperative outcomes. The BAS procedure, as recommended by a pediatric cardiologist following an echocardiographic evaluation, was performed in 55.42% of cases. The BAS rate was higher than that reported in a recent study (27.7%) [40]. In eight cases (17.39%), BAS was indicated even before birth by measuring the FO size and flap motility [10,41]. The procedure was performed at the NICU bedside in 47% of cases and the neonatal cardiac surgery unit in 53% of cases, about 8.71 h after birth on average. Umbilical venous access was utilized in 73% of cases, while femoral access was used in 27% of cases. All patients undergoing BAS were sedated, intubated, and mechanically ventilated during the procedure. In our study, in all infants, we maintained PGE1 infusion at the lowest effective dose until the day of surgery (14.1 ± 6.6 days) following the BAS procedure. There are controversies regarding the discontinuation of PGE1 treatment once adequate mixing has been achieved at the atrial level. Thus, some studies have shown that approximately 33–50% of infants have PGE1 restarted due to the risk of rebound hypoxemia after discontinuation [42,43].

### 4.1. Cerebral Oxygenation

Many studies have defined reference ranges for crSO_2_ and cFTOE during the first few days after birth [44,45]. In full-term infants, crSO_2_ rapidly increases from 44% at 3 min after birth to between 70% and 80% at 15 min and then stabilizes. At the same time, cFTOE decreases from a median of 0.33 (0.11–0.70) at 2 min to 0.18 (0.07–0.34) at 15 min after birth [44].

In our study, treatment with PGE1 significantly increased crSO_2_ from 47% to 50% in the PGE1 group at 24 h of life. Additionally, there was an increase in SpO_2_ and pre-ductal pO_2_, while the cFTOE remained constantly elevated, likely due to an increased oxygen demand. However, these values remained below those of healthy newborns. The PGE1 treatment alone did not have the expected effect on SpO_2_, pre-ductal pO_2_, and crSO_2_ in the D-TGA group with restrictive ASD/FO. Performing BAS significantly increased these parameters due to improved mixing at the atrial level and a 12.5% rise in SpO_2_, reflected mainly in the increase in crSO_2_ at 51%. Basically, at 24 h of life, following BAS, the SpO_2_, pre-ductal pO_2_, crSO_2_, and cFTOE values reached levels like those of the group that received PGE1 alone. In a previous study, the mean crSO_2_ values were found to be 56.3 ± 11.3% [46]. Another study reported median values of 48% at 2 h after BAS and 64% at 24 h after BAS [47] in newborns with D-TGA. However, it is worth noting that the timing of measurement in these studies differed from the timing in our study.

CrSO_2_ depends on the level of oxygen delivery and brain oxygen consumption, reflecting the balance between the cerebral oxygen supply and demand [48]. Oxygen delivery is closely related to SpO_2_, the cerebral blood flow, and the hemoglobin level. However, in our study, the low crSO_2_ value appears unlikely to be caused by a decrease in the hemoglobin concentration, especially since the values at birth were within normal limits. Due to the anatomical and functional characteristics of the malformation, infants with D-TGA typically have SpO_2_ levels less than 85% and crSO_2_ in the low 50% range [49]. A reassuring SpO_2_ level between 80 and 85% obtained after PGE1 treatment and/or BAS at 24 h of life was associated with low crSO_2_, indicating low cerebral oxygen delivery or increased oxygen demands due to hypoxia. Low crSO_2_ values suggest cerebral hypoxic hypoxia, while high FTOE values suggest cerebral ischemic hypoxia, both indicating low oxygen availability to cerebral tissue. Prolonged cerebral hypoxia can lead to increased central nervous system injury, either before or after birth, particularly affecting the white matter. In our study, we did not find any cerebral lesions, but we observed that the head circumference was significantly smaller in the PGE1 + BAS group compared to the control group (33.8 ± 1.3 cm vs. 34.5 ± 1 cm, *p* = 0.0151). The study conducted by Licht found that newborns with D-TGA had a delay in brain development of approximately one month and a mean head circumference one standard deviation below the expected value for their age [50]. However, surgical intervention must be performed at the optimal time to prevent future neurological complications.

### 4.2. Cerebral Blood Flow

Adequate postnatal cerebral blood flow, which is closely related to cardiac output and cerebral vascular resistance, is essential for the delivery of oxygen to brain tissue. Newborns with D-TGA have altered cerebral hemodynamics compared to healthy newborns. Healthy newborns experience an increased cerebral blood flow within the first few weeks of life, marked by elevated flow velocities in cerebral arteries, likely caused by the closure of the arterial duct [23].

Bedside Doppler ultrasound measurements instantly provide imaging for monitoring brain perfusion, as continuous information about the cerebral blood flow is lacking. Our center performs dynamic Doppler assessments, including after adjusting PGE1 doses. Our study revealed that PGE1 treatment resulted in a significant increase in the RI from birth to 24 h after delivery in the group treated with PGE1 alone due to a decrease in EDV, while PSV remained unchanged. The increase in RI was even more pronounced in the group that underwent septostomy, both before and after the procedure, respectively, at 24 h after delivery. We consider that cerebral perfusion is diminished due to the ductus arteriosus maintained open by PGE1 treatment. The phenomenon of ductal stealing in the case of a large patent ductus arteriosus may alter the cerebral hemodynamics.

The existing literature offers limited information on pre-surgery Doppler measurements of the cerebral arteries in newborns with D-TGA. Mir et al. monitored 38 patients with congenital heart disease using cerebral Doppler ultrasound. They found increased PSV, decreased end EDV (β = −5.75 cm/s, 95% CI −8.38 to −3.12, *p* < 0.001), and a significant increase in the resistive index of the anterior cerebral artery (ACA) (β = 0.16, 95% CI 0.10–0.22, *p* < 0.001). There was no retrograde diastolic flow in the ACA [51]. In a prospective study of pediatric patients undergoing congenital heart surgery, ultrasound RI of the major cranial vessels was measured prior to surgery, immediately post-operatively, and prior to discharge. There were no significant correlations between the RI and neurodevelopmental outcomes [52].

This study has limitations due to its retrospective nature and the limited number of cases. We investigated changes in cerebral oxygenation and hemodynamics during the transition period of newborns with D-TGA based on emergency management. Larger sample sizes, provided by multicenter studies, would be more valuable. More information is needed on the impact of abnormal blood flow associated with congenital heart defects. This may help to identify the optimal timing for surgical correction and to optimize neurodevelopmental outcomes.

## 5. Conclusions

The PGE1 treatment and atrial septostomy are life-saving interventions that can improve peripheral oxygenation and cerebral perfusion shortly after initiation. This is reflected by increasing SpO_2_ and crSO_2_ in newborns with D-TGA during their transition to extrauterine life, although these values are below the average. PGE1 administration significantly decreased end-diastolic velocities in the anterior cerebral artery but did not affect systolic velocities. Both PGE1 treatment and atrial septostomy are only temporary measures until surgical correction is performed. If low crSO_2_ is detected despite PGE1 administration, atrial septostomy is necessary. Similarly, if an abnormal diastolic flow is detected, indicating an impaired cerebral blood flow, early surgical correction is needed to prevent cerebral hypoxia–ischemia.

## Figures and Tables

**Table 1:** Timing of patient evaluation.

Time	PGE1 Group	PGE1 + BAS Group	Control Group
1.	Echocardiogram	Echocardiogram	-
	CDU (PSV, EDV, and RI)	CDU (PSV, EDV, and RI)	CDU (PSV, EDV, and RI)
	crSO_2_ and cFTOE	crSO_2_ and cFTOE	crSO_2_ and cFTOE
	Pre-ductal SpO_2_	Pre-ductal SpO_2_	Pre-ductal SpO_2_
	BP (systolic/diastolic/MAP)	BP (systolic/diastolic/MAP)	BP (systolic/diastolic/MAP)
	FiO_2_	FiO_2_	FiO_2_
	pH, pO_2_, and lactate	pH, pO_2_, and lactate	pH, pO_2_, and lactate
2.	-	Echocardiogram	-
	-	CDU (PSV, EDV, and RI)	-
	-	crSO_2_ and cFTOE	-
	-	Pre-ductal SpO_2_	-
	-	BP (systolic/diastolic/MAP)	-
3.	Echocardiogram	Echocardiogram	
	CDU (PSV, EDV, and RI)	CDU (PSV, EDV, and RI)	CDU (PSV, EDV, and RI)
	crSO_2_ and cFTOE	crSO_2_ and cFTOE	crSO_2_ and cFTOE
	Pre-ductal SpO_2_	Pre-ductal SpO_2_	Pre-ductal SpO_2_
	BP (systolic/diastolic/MAP)	BP (systolic/diastolic/MAP)	BP (systolic/diastolic/MAP)
	FiO_2_	FiO_2_	FiO_2_
	pH, pO_2_, and lactate	pH, pO_2_, and lactate	pH, pO_2_, and lactate

Time 1—within 2 h of life; Time 2—before BAS procedure; Time 3—at 24 h of life; PGE1—prostaglandin E1; BAS—balloon atrial septostomy; CDU—Doppler cerebral ultrasound; PSV—peak systolic velocity; EDV—end-diastolic velocity; RI—resistive index; crSO_2_—cerebral regional tissue oxygen saturation; cFTOE—cerebral fractional tissue extraction; SpO_2_—peripheral oxygen saturation; BP—blood pressure; MAP—mean arterial pressure; FiO_2_—fraction of inspired oxygen; pO_2_—partial pressure of oxygen.

**Table 2:** Baseline characteristics.

Demographic Variables	Study Group		Control Group *n* = 77	*p*-Value PGE1 vs. PGE1 + BAS	*p*-Value PGE1 vs. Control	*p*-Value PGE1 + BAS vs. Control
	PGE1 Group *n* = 37	PGE1 + BAS Group *n* = 46				
GA ^§^, weeks, mean (SD)	39 (1.5)	39 (1.3)	39.4 (1.1)	0.9970	0.3691	0.3301
BW ^§^, g, mean (SD)	3292.4 (528.5)	3302 (442.8)	3380.1 (467.9)	0.9245	0.3580	0.3817
HC ^§^, cm, mean (SD)	34 (1.4)	33.8 (1.3)	34.5 (1.0)	0.8619	0.1085	0.0151 *
Antenatal care ^‡^, *n* (%)	30 (81.1)	42 (91.3)	61 (79.2)	0.1721	0.8167	0.0788
Prenatal diagnosis ^‡^, *n* (%)	26 (70.3)	29 (63.04)	-	0.7869	-	-
In utero transfer ^‡^, *n* (%)	19 (51.4)	18 (39.1)	-	0.5377	-	-
Cesarian delivery ^‡^, *n* (%)	18 (48.7)	27 (58.7)	27 (35.1)	0.3611	0.1647	0.0106 *
Apgar 1 min ^§^, median (IQR)	8 (8–9)	8 (8–8)	10 (9–10)	0.9912	<0.001 *	<0.001 *
Apgar 5 min ^‡^ median (IQR)	8 (8–9)	8 (8–9)	10 (10–10)	0.9991	<0.001 *	<0.001 *
Age at surgery (days) ^§^, mean (SD)	14.8 (10.1)	14.1 (6.6)	-	0.6865	-	-
Deaths ^‡^, *n* (%)	1 (2.7)	8 (17.4)	-	0.0240 *	-	-
Pre-surgery death ^‡^, *n* (%)	0 (0)	3 (6.5)	-	0.1627	-	-
Post-surgery death ^‡^, *n* (%)	1 (2.7)	5 (10.8)	-	0.0715	-	-

Continuous variables were expressed as median (IQR—interquartile range) when their distribution was not normal and as mean (SD—standard deviation) when the variables had a normal distribution. Categorical variables were expressed as number (%), GA—gestational age, BW—birth weight, and HC—head circumference. ^§^ Mann–Whitney U test or Student’s *t*-test. ^‡^ Pearson chi-square test. (*) Marked effects are significant at *p* < 0.05.

**Table 3:** Clinical characteristics, interventions, and heart ultrasound evaluation.

	PGE1 Group (*n* = 37)	PGE1 + BAS Group (*n* = 46)	*p*-Value
1. Clinical characteristics			
Tachypnea ^‡^, *n* (%)	24 (64.9)	38 (82.6)	0.0645
Cyanosis ^‡^, *n* (%)	37 (100)	46 (100)	0.9999
Cardiac murmur (grade) ^§^, median (IQR)	3 (2–3)	3 (2–3)	0.4134
Need for MV ^§^, mean (SD)	11 (29.7)	23 (50)	0.0619
Need for inotrope ^§^, mean (SD)	14 (37.8)	18 (39.1)	0.9042
2. Interventions			
PGE1 doses (μg/kg/min) ^§^, mean (SD)			
Initial ^§^, mean (SD)	0.042 (0.024)	0.050 (0.025)	0.1440
Before BAS ^§^, mean (SD)	-	0.054 (0.019)	-
At 24 h of life ^§^, mean (SD)	0.040 (0.016)	0.031 (0.015)	0.0136 *
Time to BAS (hours after birth) ^§^, mean (SD)	-	8.71 (5.05)	-
3. Heart ultrasound evaluation			
PDA			
Size at birth (mm) ^§^, mean (SD)	4.08 (1.31)	3.74 (1.00)	0.1929
Left–Right flow ^‡^, *n* (%)	22 (59.5)	21 (45.7)	0.2108
Right–Left flow ^‡^, *n* (%)	1 (2.70)	1 (2.71)	0.8759
Bidirectional flow ^‡^, *n* (%)	16 (43.24)	24 (52.17)	0.4183
ASD/PFO			
Size ASD at birth (mm) ^§^, median (IQR)	4.8 (4.0–5.2)	3 (2.5–3.3)	<0.001 *
Size after BAS (mm) ^§^, median (IQR)	-	4.65 (4.1–5.2)	-
Restrictive < 4 mm ^‡^, at birth *n* (%)	0 (0)	46 (100)	<0.001 *
Gradient (mmHg) ^§^, median (IQR)	5 (3–6.6)	8.7 (7.7–11)	<0.001 *
VSD (mm) ^§^, mean (SD)	1.57 (2.59)	0.63 (1.99)	0.0393 *
Number of cardiac ultrasound examinations ^§^, median (IQR)	7 (6–9)	7 (6–8)	0.7382

Continuous variables were expressed as median (IQR—interquartile range) when their distribution was not normal and as mean (SD—standard deviation) when the variables had a normal distribution. Categorical variables were expressed as number (%); MV—mechanical ventilation; PGE1—prostaglandin E1; BAS—balloon atrial septostomy; PDA—patent ductus arteriosus; ASD/PFO—atrial septal defect/persistent foramen ovale; VSD—ventricular septal defect. ^§^ Mann–Whitney U test or Student’s *t*-test. ^‡^ Pearson chi-square test. (*) Marked effects are significant at *p* < 0.05.

**Table 4:** Cerebral oxygenation and cerebral blood flow evaluation.

Clinical Variables	Study Group		Control Group *n* = 77	*p*-Value PGE1 vs. BAS	*p*-Value PGE1 vs. Control	*p*-Value PGE1 + BAS vs. Control
	PGE1 Group *n* = 37	PGE1 + BAS Group *n* = 46				
crSO_2_ ^§^, (%) median (IQR)						
within 2 h of life (before PGE1)	47 (41–55)	43 (39–47)	83 (78–86)	0.0412 *	<0.001 *	<0.001 *
before BAS	-	42 (39–46)	-			
24 h of life	50 (47–54)	51 (48–59)	87 (84–91)	0.2096	<0.001 *	<0.001 *
cFTOE ^§^, median (IQR)						
within 2 h of life (before PGE1), mean (SD)	0.38 (0.34–0.4)	0.39 (0.33–0.45)	0.16 (0.12–0.21)	0.8938	<0.001 *	<0.001 *
before BAS		0.45 (0.35–0.48)				
24 h of life	0.38 (0.33–0.43)	0.38 (0.31–0.42)	0.11 (0.08–0.15)	0.8240	<0.001 *	<0.001 *
SpO_2_ pre-ductal (%) ^§^, median (IQR)						
within 2 h of life (before PGE1)	80 (75–83)	72 (65–78)	99 (98–100)	0.00012 *	0.00002 *	0.00002 *
before BAS		77 (72–80)				
24 h of life	82 (80–85)	84.5 (83–87)	99 (98–100)	0.00003 *	0.00002 *	0.00002 *
RI ^§^, mean (SD)						
within 2 h of life (before PGE1)	0.718 (0.054)	0.708 (0.054)	0.713 (0.05)	0.3990	0.6812	0.5761
before BAS		0.759 (0.046)				
24 h of life	0.769 (0.036)	0.764 (0.041)	0.681 (0.046)	0.6272	0.00002 *	0.00002 *
PSV ^§^, (cm/s), mean (SD)						
within 2 h of life (before PGE1)	39.75 (7.41)	37.78 (7.82)	34.49 (6.71)	0.4177	0.0007 *	0.03831
before BAS		40.41 (8.92)				
24 h of life	38.99 (5.29)	39.72 (6.53)	37.35 (7.80)	0.8958	0.5651	0.2313
EDV ^§^, (cm/s), mean (SD)						
within 2 h of life (before PGE1)	10.71 (1.61)	10.86 (2.54)	9.78 (2.01)	0.7171	0.0292 *	0.0153 *
before BAS		9.27 (1.91)				
24 h of life	8.74 (1.31)	9.16 (2.00)	11.83 (2.39)	0.687	0.00001 *	0.00002 *
pH ^§^, mean (SD)						
within 2 h of life (before PGE1)	7.29 (0.09)	7.29 (0.07)	7.34 (0.05)	0.6967	0.0043 *	0.0054 *
before BAS		7.31 (0.11)				
24 h of life	7.35 (0.08)	7.35 (0.06)	7.38 (0.03)	0.872	0.0211 *	0.03638 *
pO_2_ ^§^ (mmHg), median (IQR)						
within 2 h of life (before PGE1)	31 (28–34)	26 (21–29)	31 (28–36)	0.0431	0.8072	0.2038
before BAS		25.60 (7.06)				
24 h of life	39 (35–41)	38 (29–46)	56 (51–61)	0.9879	<0.001 *	<0.001 *
Lactate ^§^ (mmol/L), median (IQR)						
within 2 h of life (before PGE1)	3.2 (2.4–4.1)	3.05 (2–4.7)	2.9 (1.9–3.7)	0.0499	0.5567	0.1385
before BAS		4.60 (2.30)				
24 h of life	2.2 (1.9–3.1)	2.15 (1.5–4.5)	2.1 (1.8–2.4)	0.9818	0.2498	0.3138
Hgb ^§^ (g/dL), median (IQR)						
within 2 h of life (before PGE1)	16.3 (15.6–16.7)	15.9 (15.3–17.2)	17.7 (16.7–18.3)	0.9902	<0.0001 *	<0.0001 *
Mean BP (MAP) ^§^, (mmHg), mean (SD)						
within 2 h of life (before PGE1)	43.73 (9.18)	49.32 (7.83)	47.47 (7.12)	0.0012 *	0.0189 *	0.2429
before BAS		45.41 (5.67)				
24 h of life	48.59 (6.76)	50.67 (6.34)	52.22 (7.14)	0.1338	0.0242 *	0.2648
Systolic BP ^§^, (mmHg), mean (SD)						
within 2 h of life (before PGE1)	65.41 (11.44)	65.54 (7.19)	67.40 (9.15)	0.9413	0.3187	0.3214
before BAS		63.87 (9.01)				
24 h of life	65.41 (11.44)	69.78 (7.98)	73.10 (7.86)	0.0147 *	0.00003 *	0.0642
Diastolic BP ^§^, (mmHg), mean (SD)						
within 2 h of life (before PGE1)	29.70 (10.07)	33.67 (7.05)	36.96 (7.32)	0.0140 *	0.00004 *	0.0283 *
before BAS		29.93 (6.32)				
24 h of life	31 (26–34)	33 (29–37)	39 (34–47)	0.5780	<0.0001 *	<0.0001 *
FiO_2_ (%) ^§^, median (IQR)						
within 2 h of life (before PGE1)	30 (21–40)	40 (30–60)	21 (21–21)	0.1006	<0.0001 *	<0.0001 *
before BAS		40 (30–60)				
24 h of life	21 (21–30)	23 (21–30)	21 (21–21)	0.9981	0.0008 *	<0.0001 *

Continuous variables were expressed as median (IQR—interquartile range) when their distribution was not normal and as mean (SD–standard deviation) when the variables had a normal distribution. Categorical variables were expressed as number (%); PGE1—prostaglandin E1; BAS—balloon atrial septostomy; crSO_2_—cerebral regional tissue oxygen saturation; cFTOE—cerebral fractional tissue extraction; RI—resistive index; PSV—peak systolic velocity; EDV—end-diastolic velocity; Hgb—hemoglobin; BP—blood pressure; FiO_2_—fraction of inspired oxygen. ^§^ Mann–Whitney U test or Student’s *t*-test. (*) Marked effects are significant at *p* < 0.05.

**Table 5:** Comparison of cerebral/peripheric oxygenation and cerebral velocities between groups.

Clinical Variables	PGE1 Group *n* = 37			PGE1, BAS + Group *n* = 46			
	2 h of Life	24 h of Life (after PGE)	*p*-Value	2 h of Life	Before BAS	24 h of Life (after BAS)	*p*-Value
crSO_2_ ^§^	47 (41–55)	50 (47–54)	0.00029 *	43 (39–47)	42 (39–46)	51 (48–59)	<0.0001 *
cFTOE ^§^	0.38 (0.34–0.4)	0.38 (0.33–0.43)	0.1717 *	0.39 (0.33–0.45)	0.45 (0.35–0.48)	0.38 (0.31–0.42)	0.0002 *
SpO_2_ preductal ^§^	80 (75–83)	82 (80–85)	0.0053 *	72 (65–78)	77 (72–80)	84.5 (83–87)	<0.0001 *
RI ^§^	0.718 (0.054)	0.769 (0.036)	0.000002 *	0.708 (0.054)	0.759 (0.046)	0.764 (0.041)	0.00002 *
PSV ^§^	39.75 (7.41)	38.99 (5.29)	0.2512	37.666 (7.82)	40.41 (8.92)	39.72 (6.53)	0.49415
EDV ^§^	10.71 (1.61)	8.74 (1.31)	<0.0001 *	10.86 (2.54)	9.27 (1.91)	9.16 (2.00)	0.00004 *

Continuous variables were expressed as median (IQR—interquartile range) when their distribution was not normal and as mean (SD–standard deviation) when the variables had a normal distribution. Categorical variables were expressed as number (%); PGE1—prostaglandin E1; BAS—balloon atrial septostomy; crSO_2_—cerebral regional tissue oxygen saturation; cFTOE—cerebral fractional tissue extraction; RI—resistive index; PSV—peak systolic velocity; EDV—end-diastolic velocity; Hgb—hemoglobin; BP—blood pressure; FiO_2_—fraction of inspired oxygen. ^§^ Mann–Whitney U test or Student’s *t*-test. (*) Marked effects are significant at *p* < 0.05.

## Data Availability

The data presented in this study are available on request from the corresponding authors.
